# Cost-effectiveness of transcatheter aortic valve implantation for asymptomatic severe aortic stenosis in the Kingdom of Saudi Arabia

**DOI:** 10.3389/fcvm.2026.1937664

**Published:** 2026-10-29

**Authors:** Hussain Abdulrahman Al-Omar, Madison Montgomery, Heather Davies, Elana Greaves, Reagan Davis, Lucy Hillcoat, Hazim J. Rahbi, Adham Salem, Pascal Candolfi, Abdulaziz Al Rabiah

**Affiliations:** 1 Department of Clinical Pharmacy, College of Pharmacy, King Saud University, Riyadh, Saudi Arabia; 2 York Health Economics Consortium Ltd, Heslington, United Kingdom; 3 Heart Health Center, King Saud Medical City, Riyadh First Health Cluster, Riyadh, Saudi Arabia; 4 Edwards Lifesciences, Dubai, United Arab Emirates; 5 Edwards Lifesciences, Nyon, Switzerland; 6 Saudi Arabia Ministry of Health, Riyadh, Saudi Arabia

**Keywords:** asymptomatic severe aortic stenosis, clinical surveillance, cost-effectiveness, early intervention, Kingdom of Saudi Arabia, transcatheter aortic valve implantation

## Abstract

**Introduction:**

Aortic stenosis (AS) is defined as the narrowing of the aortic valve. The benefits of implementing early aortic valve replacement (AVR), while the AS is asymptomatic, have been demonstrated in various clinical trials compared with clinical surveillance (CS) of delayed AVR upon symptom onset. The study aims to estimate the cost-effectiveness of transcatheter aortic valve implantation (TAVI) for the treatment of asymptomatic severe aortic stenosis (aSAS) in the Kingdom of Saudi Arabia (KSA).

**Methods:**

A cost–utility analysis compared TAVI using the SAPIEN 3/SAPIEN 3 Ultra valve with CS in an aSAS population. A Markov model consisting of three health states—alive and well, stroke, and death—captured the peri-procedural and long-term outcomes across a lifetime horizon. Inputs were derived from the EARLY TAVR trial (NCT03042104) and existing literature.

**Results:**

The model findings estimated that TAVI was cost-saving and more effective (dominant) compared with CS for people with aSAS, with a 97.3% probability of being cost-effective. Incremental results per person included an estimated cost saving of SAR 33,414, an estimated quality-adjusted life year gain of 0.17, and an estimated life-year gain of 0.12. The key driver of the model was the weighting of disabling stroke for the CS arm.

**Conclusion:**

Early implementation of TAVI for those with aSAS is estimated to result in reduced costs and greater benefits compared with CS. These findings support the idea that timely intervention could enhance AS disease management in line with KSA policy.

## 1 Introduction

Aortic stenosis (AS) is a prevalent valvular heart disease characterised by the narrowing of the aortic valve and is associated with an estimated global prevalence of 5% in individuals over 65 years (1, 2). AS is associated with increased morbidity and mortality, further exacerbated by AS severity and symptom progression (3–5). For example, when left untreated, symptomatic severe AS (sSAS) is associated with a 60%–90% increase in mortality over 5 years, whereas the asymptomatic severe (aSAS) form of the disease is associated with a 44% increased risk of mortality over 4 years (6, 7).

Current standard of care for sSAS includes aortic valve replacement (AVR), a procedure that is performed via traditional surgery (surgical aortic valve replacement, SAVR) or via a transcatheter approach (transcatheter aortic value implantation, TAVI). However, approximately one-third of individuals with AS are diagnosed with aSAS (8). This population has historically remained under clinical surveillance (CS) until symptom onset or decreased systolic function (9–13).

A longer time to intervention has been associated with a progressive increase in healthcare costs in a symptomatic population (14). There has also been a growing body of clinical evidence establishing the potential benefits of performing early AVR instead of conservative measures in an older aSAS population, including a noticeable reduction in heart failure-related hospitalisation (HFH), stroke, and myocardial infarction (15–17). In particular, a recent meta-analysis examining four randomised controlled trials (RCTs) (*n* = 1,427) observed significant reductions in HFH [Hazard Ratio (HR): 0.31] and cardiovascular mortality (HR: 0.56). This research contributed to updates of the 2025 European Guidelines for the Management of Valvular Heart Disease, which endorse early AVR for aSAS if procedural risk is low (Class 2a, Level A) (18). Although the Kingdom of Saudi Arabia (KSA) has dedicated guidelines for the clinical management of AS, clinicians in the KSA often follow the European guidelines because they are internationally recognised and incorporate more recent evidence and treatment modalities (18, 19). The KSA guidelines do not yet endorse TAVI for aSAS, as the guidelines were published before the conclusion of the EARLY TAVR trial. However, recent national KSA policy has called for a strategic initiative to raise awareness, enhance timely diagnosis, and improve the management of valvular heart disease (20).

The EARLY TAVR (NCT03042104) RCT recruited 901 individuals with aSAS to receive either CS or early TAVI (with SAPIEN 3/SAPIEN 3 Ultra valves) (21). Over a median follow-up of 3.8 years, TAVI demonstrated a 0.8% reduction in death, a 2.5% reduction in stroke, and a 20.8% reduction in unplanned cardiovascular hospitalisations compared with CS, suggesting that early TAVI is superior (22). Although the clinical efficacy and cost-effectiveness of TAVI compared with CS have been explored, the cost-effectiveness has yet to be established in the KSA healthcare system setting (23, 24). Therefore, we aimed to determine the cost-effectiveness of TAVI compared with CS for aSAS in the KSA using data from the EARLY TAVR RCT.

## 2 Methods

### 2.1 Model overview

The cost-effectiveness of TAVI was modelled from a KSA healthcare system perspective. As no KSA-specific health technology assessment (HTA) guidelines were available to inform the discount rates and the willingness-to-pay (WTP) threshold, alternative sources were identified (25–30). The discount rates for costs and benefits were set equal to those recommended by the National Institute for Health and Care Excellence (NICE) in their reference case: 3.5% per year (25). The WTP threshold was informed by the gross domestic product per capita reflective of the 2024 cost year and was calculated to be SAR 131,706 (26, 27).

Both economic and clinical outcomes were captured in this analysis. The economic outcomes included total mean per person costs, life years and quality-adjusted life years (QALYs), the incremental cost-effectiveness ratio (ICER), and net monetary benefit. The cost outcomes were reported in both SAR and US dollars, using a conversion rate of 1.00 SAR to 0.26̅ USD (31). The Consolidated Health Economic Evaluation Reporting Standards (CHEERS) guidelines informed the reporting standards for this analysis (32).

### 2.2 Model structure

The model in this study was adapted from existing analyses that used a Markov model to investigate the cost-effectiveness of TAVI compared with CS for aSAS in the United States (US) and across nine European countries, with validation by a key opinion leader (KOL) in each country (23, 24).

The overall model structure remained the same for the KSA adaptation and was validated by an interventional cardiology expert based in the KSA (Figure 1). To appropriately capture the peri-procedural events and longer-term impacts of TAVI, a 30-day cycle length was used across a lifetime horizon. The model was built in Microsoft Excel and consisted of three health states: alive and well, stroke, and death. The aSAS cohort entered the model in the alive-and-well health state and was allocated to either the TAVI or CS model arm. In the CS arm, individuals received CS until symptom progression, at which point they would receive AVR, either surgically or via a transcatheter. A risk of stroke (disabling or non-disabling) was applied to individuals in both arms, with a simplifying assumption that an individual can have only one stroke per lifetime. This was deemed conservative due to the EARLY TAVR trial indicating higher instances of stroke in the CS arm. Death was an absorbing state: The cohort could progress to this state at any point and would remain there until the end of the time horizon. The model also included a tunnel state reflecting unplanned HFH. The cohort could enter the tunnel state at any point for one cycle and could experience an unlimited number of HFHs. Existing cost-effectiveness models that have explored TAVI for individuals with low-risk sSAS used similar methodologies which align with those used in this model (33–41). A more detailed description of the model structure is available in Généreux et al. (23).

**Figure 1:**
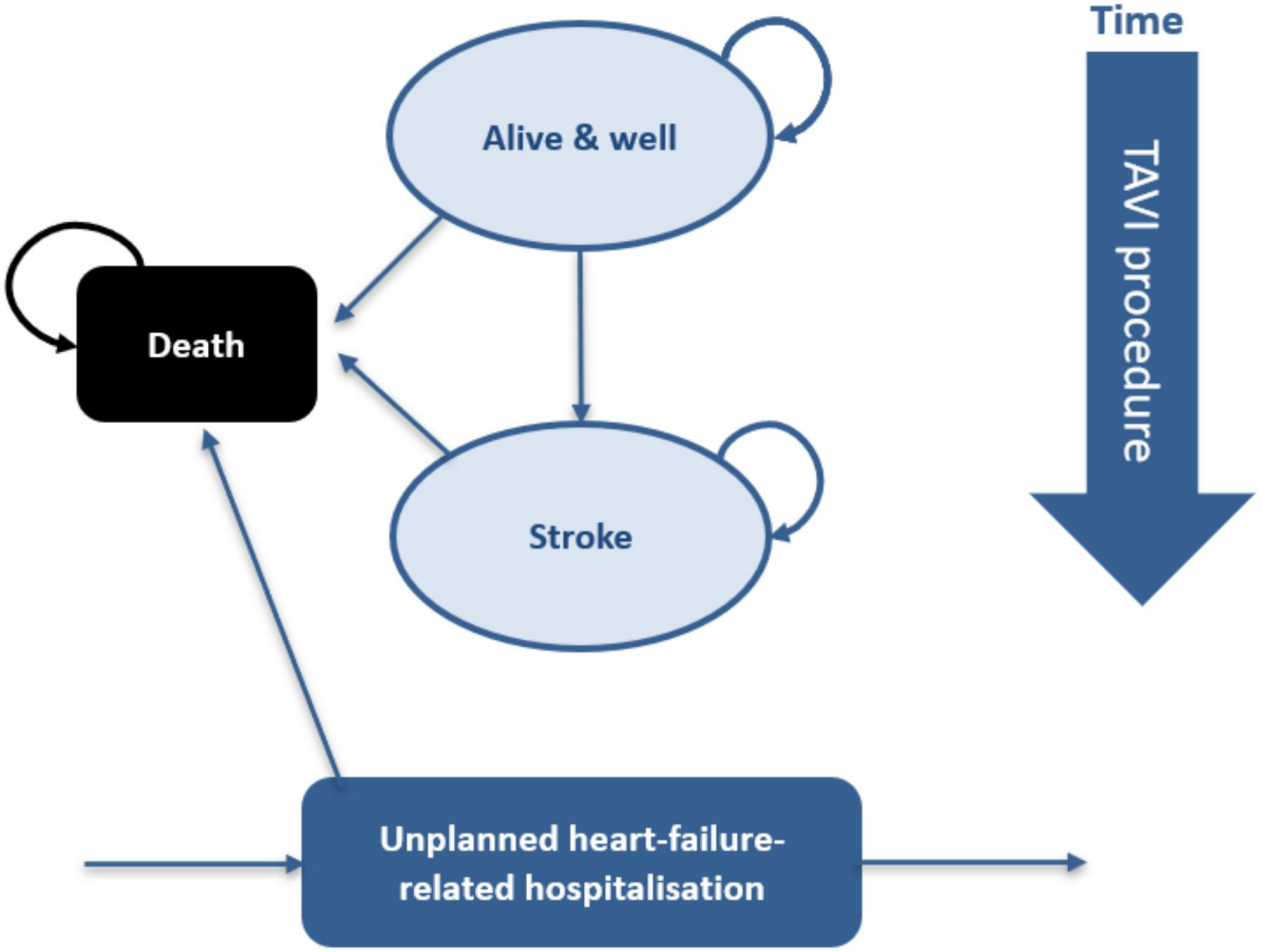
Model structure. Individuals in the TAVI arm begin in the alive-and-well health state and undergo TAVI in the first model cycle. The clinical surveillance arm can undergo aortic valve replacement at any point (upon symptom onset). Each cycle, a percentage of the cohort will experience a stroke, which can be disabling or non-disabling. At any point, an unplanned heart failure-related hospitalisation can be experienced regardless of health state allocation. This figure was adapted from Généreux et al. (23). TAVI, transcatheter aortic valve implantation.

### 2.3 Model inputs

#### 2.3.1 Clinical inputs

The EARLY TAVR trial was used as the primary source for clinical inputs (22). Baseline characteristics of the modelled cohort were informed by the EARLY TAVR trial, reflecting a starting age of 75.8 years, with 69.1% of individuals being male (22).

The TAVI cohort received AVR in the first model cycle (22). In comparison, the time to delayed AVR in the CS arm was determined using time-to-event trial data, extrapolated beyond the trial period using exponential distribution (Supplementary Figure 1, Supplementary Table 1) (42). In the CS arm, 1.8% of individuals received SAVR as opposed to TAVI. Due to the extrapolation, all individuals in the alive-and-well health state received AVR within the model time horizon.

Probabilities pertaining to annual strokes and HFHs were obtained from EARLY TAVR data and digitised using WebPlotDigitizer (v5) (23, 24, 42). Beyond the 5-year trial data, the probability of having a stroke or HFH was assumed to be the same as in year 5 to remain consistent with previous publications (23, 24). Consistent with the EARLY TAVR trial data, 42.1% of strokes were disabling in the TAVI arm and 43.3% of strokes were disabling in the CS arm (22). Further, data pertaining to cardiovascular hospitalisation was captured in the EARLY TAVR trial, specifically subsequent AVR within 6 months of randomisation for the CS arm (22). AVR is captured separately in the model. Therefore, HFH rates were used instead to attenuate the risk of double counting. The annual event rates were converted to probabilities for use in the economic model (Table 1).

**Table 1:** Key clinical inputs and mortality inputs.

Parameter	TAVI	CS	Source
Proportion of strokes that are disabling	42.1%	43.3%	Généreux et al. (22)
Proportion receiving a permanent pacemaker	5.7%	8.4%	Généreux et al. (22)
Stroke probability: year 1	1.31%	1.78%	Généreux et al. (22)^a^
Stroke probability: year 2	1.33%	2.03%	Généreux et al. (22)^a^
Stroke probability: year 3	1.35%	1.85%	Généreux et al. (22)^a^
Stroke probability: year 4	0.68%	1.88%	Généreux et al. (22)^a^
Stroke probability: year 5+	0.92%	2.16%	Généreux et al. (22)^a^
HFH probability: year 1	1.31%	5.03%	Généreux et al. (22)^a^
HFH probability: year 2	0.22%	2.80%	Généreux et al. (22)^a^
HFH probability: year 3	1.33%	1.45%	Généreux et al. (22)^a^
HFH probability: year 4	1.35%	0.98%	Généreux et al. (22)^a^
HFH probability: year 5+	1.37%	2.22%	Généreux et al. (22)^a^
All-cause mortality	Variable by age	Variable by age	General Authority for Statistics (48), >65+ doubling rate applied (48, 49)
Disabling stroke (month 1) mortality relative risk	5.22	5.22	Myat et al. (69)
Non-disabling stroke (month 1) mortality relative risk	5.22	5.22	Myat et al. (69)
Disabling stroke (month 2+) mortality relative risk	1.58	1.58	Myat et al. (69)
Non-disabling stroke (month 2+) mortality relative risk	1.58	1.58	Myat et al. (69)
HFH mortality relative risk	1.00	1.00	Assumed equal to the general population

CS, clinical surveillance; HFH, heart failure-related hospitalisation; PSA, probabilistic sensitivity analysis; TAVI, transcatheter aortic valve implantation.

a All rates were converted to probabilities for use in the economic model.

Implantation of a new permanent pacemaker was the predominant peri-procedural event, impacting 5.7% of the TAVI arm and 8.4% of the CS arm. The rate of permanent pacemaker implantation was applied to all individuals receiving AVR (regardless of health state) because it was assumed that a permanent pacemaker would be implanted during an AVR procedure and would not impact mortality risk (Table 1).

It was assumed that AVR reintervention could not occur in the same cycle in which AVR was received. For the TAVI arm, the probability of reintervention was derived from the 1- to 7-year outcomes from the PARTNER 3 trial, a study that investigated TAVI compared with SAVR in a low-risk sSAS population (43, 44). The probability of reintervention at year 8 was assumed to be equivalent to year 7, and year 9 onwards was informed by Bourguignon et al. (44, 45). We performed a scenario analysis to explore the impact of using alternative data sources for the reintervention probability (46, 47). A constant, average reintervention probability of 0.13% was applied per cycle for the CS cohort, implemented due to structural limitations within the model. In particular, the memoryless nature of the Markov model limited the traceability of cycles passed since receiving an index AVR. Therefore, it was assumed that one probability (0.13%) would be applied in all years. The annual probabilities for the TAVI arm are presented in Supplementary Table 2.

#### 2.3.2 Mortality

Health-state-specific relative risks were applied to general population mortality for the KSA population, an approach consistent with previous models (33–39, 48) (Table 1). As the alive-and-well state reflected an asymptomatic population, an additional mortality risk was not applied. Considering that the impact of a stroke event on mortality was deemed greater than experiencing a HFH, it was assumed that there would be no increase in mortality for those temporarily entering the HFH tunnel state.

The KSA life tables provide the same average mortality for those aged ≥65 years (48). In the base case, aggregated KSA-specific general population mortality data up to the age of 65 years were applied, with a non-linear extrapolation approach applied (Gompertz distribution with a mortality doubling time of 7 years) to derive rates for those above 65 years (48, 49). This method was used to increase granularity and to mitigate underestimation of mortality. A scenario using the KSA mortality data without extrapolation was conducted to observe any differences or impact on the model results. EARLY TAVR trial data were considered for mortality. However, the trial reported higher survival rates for the aSAS population than the general population. Therefore, these data were used in scenario analyses rather than the base case.

#### 2.3.3 Costs and resource use

Cost data were predominantly derived from Carapinha et al. and were inflated (where appropriate) to reflect the 2024/25 cost year (50, 51). Other inputs, such as HFH-associated costs, were sourced from the KSA Ministry of Health costing data and stakeholder opinion (52). Due to a paucity of KSA-specific data, the cost assigned to a non-disabling stroke was adjusted from a United Kingdom (UK) benchmark to KSA using a purchasing power parity factor (53, 54). The UK value was sourced from National Health Service cost collection data (55). The cost inputs are presented in Table 2.

**Table 2:** Other model inputs.

Parameter	Value	Source
Echocardiogram (unit cost)	SAR 1,000	Saudi Arabia Ministry of Health services costing directory (52)
Consultation (unit cost)	SAR 350	Saudi Arabia Ministry of Health services costing directory (52)
Disabling stroke (initial cost)	SAR 12,169	Carapinha et al. (50)
		Inflated using World Bank Group (51)
Disabling stroke (ongoing cost per year)	SAR 207,452	Carapinha et al. (50)
		Inflated using World Bank Group (51)
Non-disabling stroke (initial cost)	SAR 7,003	NCC (2024): weighted average, total HRGs, stroke with CC < 10, AA35D-F (55)
		Converted using World Bank Group (53, 54)
Non-disabling stroke (ongoing cost per year)	SAR 0	Assumption
HFH general ward stay	SAR 350	Saudi Arabia Ministry of Health services costing directory (52)
HFH ICU/CCU stay	SAR 1,750	Saudi Arabia Ministry of Health services costing directory (52)
HFH consultation	SAR 350	Saudi Arabia Ministry of Health services costing directory (52)
HFH CVI consultation	SAR 350	Clinical opinion
HFH: MCS procedure	SAR 128,000	National Unified Procurement Company (2025)
PPM (implantation cost)	SAR 5,793	Carapinha et al. (50)
		Inflated using World Bank Group (51)
PPM (ongoing cost per month)	SAR 1,272	Carapinha et al. (50)
		Inflated using World Bank Group (51)
TAVI: Cost of intervention	SAR 30,667	Carapinha et al. (50)
		Inflated using World Bank Group (51)
TAVI: Cost of valve	SAR 127,761	National Unified Procurement Company Price (2025)
SAVR: Cost of intervention (complex)	SAR 96,424	Carapinha et al. (50)
		Inflated using World Bank Group (51)
Echocardiogram (per year)	3	Clinical opinion
Consultation (per year)	1	Clinical opinion
HFH general ward stay (length of stay, days)	2	Clinical opinion
HFH CCU stay (length of stay, days)	7	Clinical opinion
HFH cardiology consultation	9	Clinical opinion
HFH CVI consultation	1	Clinical opinion
HFH MCS procedure (proportion requiring)	7.5%	Clinical opinion
Alive-and-well utility input	0.78	Alava et al. (57) Age and gender adjusted
Non-disabling stroke utility input	0.73	Luengo-Fernandez et al. (70)
Disabling stroke (month 1) utility input	0.13	Luengo-Fernandez et al. (70)
Disabling stroke (month 2+) utility input	0.45	Luengo-Fernandez et al. (70)
HFH utility input	0.78	Assumed to be the same as “alive-and-well”

CC, comorbidities and complications; CCU, critical care unit; CVI, cardiovascular imaging; HFH, heart failure-related hospitalisation; HRG, Healthcare Resource Group; MCS, mechanical circulatory support; NCC, National Cost Collection; NICE, National Institute for Health and Care Excellence; PPM, permanent pacemaker; PSA, probabilistic sensitivity analysis; SAR, Saudi Arabia Riyals; SAVR, surgical aortic valve replacement; TAVI, transcatheter aortic valve implantation.

The cost of TAVI included any procedural costs and the cost of the SAPIEN 3/SAPIEN 3 Ultra valve (21). The cost applied to SAVR was informed by existing literature and inflated to reflect the current cost year. Further, SAVR was applied as a complex procedure reflective of trial data (22). Consistent with existing health economic models, and due to identical hardware pricing and procedural requirements, the procedural costs of subsequent valve-in-valve reinterventions were assumed equal to the index procedure (23, 24).

Annual monitoring costs (including three echocardiograms and one consultation, validated by an interventional cardiology expert based in the KSA) were only incurred by the CS cohort in the alive-and-well health state and were applicable until AVR was received. The monitoring costs were not applicable in the TAVI arm because the procedure was performed in the first model cycle (36). HFH event costs captured the following: a 7-day visit to an intensive care unit/critical care unit ward; a 2-day visit to a general ward; nine cardiology consultations; one cardiovascular imaging consultation; and 7.5% receiving a mechanical circulatory support procedure. These events were incurred within the tunnel state (33, 37, 52).

Stroke-related costs were stratified by stroke severity and were incurred in the same cycle as the event. In addition, individuals in the disabling stroke substate accrued an ongoing cost over the time horizon.

#### 2.3.4 Health-related quality of life

Although EQ-5D-5L value sets have been published for the KSA, they have not yet been used to derive KSA-specific age-adjusted EQ-5D population norms (56). Therefore, health state-specific utility adjustment factors were multiplicatively applied to UK general population norms that were sex- and age-stratified (57, 58). The adjustment factors were calculated by dividing health state-specific utilities by age-specific utilities. Consistent with the alive-and-well state comprising an asymptomatic cohort, it was assumed that individuals in this state had a health-related quality-of-life (HRQoL) equivalent to the general population. The HFH tunnel state was not associated with a reduction in HRQoL to avoid double counting the reduced HRQoL associated with the stroke health state. This was a conservative assumption for the alive-and-well HFH substate. The utility values are presented in Table 2.

#### 2.3.5 Sensitivity analysis

To account for model uncertainty, deterministic and probabilistic sensitivity analyses were conducted. First-order parameter uncertainty was evaluated using deterministic sensitivity analysis, varying all parameters except discount rates by ±20%. The discount rates were varied between 1.5% and 6%.

Second-order parameter uncertainty was evaluated using probabilistic sensitivity analysis (PSA), by quantifying the degree of confidence in the base case results. The analysis consisted of running 1,000 iterations to determine the aforementioned certainty in results. The deterministic variance and the distributions used in the PSA for all parameters can be found in Supplementary Table 3.

Further, eight scenarios were incorporated into the model. The first scenario investigated the impact of the disabling stroke and the HFH event annual probabilities being equal to the TAVI arm for time beyond 5 years (reducing the CS arm stroke annual probability to 0.92% and the HFH annual probability to 1.37%). The second scenario investigated the proportion of people who received a new, permanent pacemaker during AVR being equal across arms (8.4%). The other six scenarios used an alternative time horizon (5 years), alternative sources for reintervention data, trial-based mortality data, life table data, and delayed AVR (6 months). The delayed AVR scenario was conducted to determine the impact on costs only due to the impact on clinical effectiveness being unknown.

To determine whether TAVI remained cost-effective when varying specific parameters, four threshold analyses were conducted. One threshold analysis was conducted for the reintervention of AVR for the CS arm. The other three analyses were conducted for the costs pertaining to the annual ongoing cost of a disabling stroke, the acute cost of a disabling stroke, and the monthly ongoing cost of a permanent pacemaker.

#### 2.3.6 Model validation

The economic model underwent a comprehensive validation process. Technical validation was conducted using a standardised health economic model review checklist to perform pressure checks and verify mathematical accuracy. Face validity of the structure, resource utilisation, and cost inputs was conducted through consultation with KOLs in the KSA. Furthermore, the model was cross validated for structural and conceptual consistency against previously published European and US economic evaluations (23, 24). Finally, external validity was assessed by comparing model-projected clinical outcomes against trial observed data from the EARLY TAVR trial, demonstrating high alignment (Supplementary Table 4).

## 3 Results

The base case indicates that, from a KSA healthcare perspective, early intervention with TAVI for people with aSAS yields lower lifetime costs (incremental: SAR −33,414) and generates more QALYs (incremental: 0.17) compared with CS (Table 3). As a result, TAVI demonstrates a dominant cost-effectiveness profile (Figure 2 and Supplementary Figure 2). These results were consistent across the majority of iterations, with TAVI demonstrating a 97.3% probability of being cost-effective. The deterministic cost breakdown is presented in Supplementary Figure 3.

**Table 3:** Model results (per person, over a lifetime time horizon).

Outcome	TAVI (probabilistic)	CS (probabilistic)	Incremental (probabilistic)	Incremental (deterministic)
Cost per person (SAR)	SAR 223,558 (CI: SAR 199,629 to 255,258)	SAR 256,972 (CI: SAR 217,853 to 306,646)	SAR −33,414 (CI: SAR −73,644 to −1,103)	SAR −33,317
Cost per person (USD)	$59,616 (CI: $53,234 to $68,069)	$68,526 (CI: 58,094 to $81,722)	−$8,910 (CI: −$19,638 to −$294)	−$8,885
QALYs per person	6.88 (CI: 5.29 to 8.37)	6.70 (CI: 5.19 to 8.13)	0.17 (CI: 0.03 to 0.37)	0.14
Life years per person	8.51 (CI: 7.37 to 9.66)	8.39 (CI: 7.31 to 9.46)	0.12 (CI: 0.00 to 0.30)	0.11
ICER, SAR per QALY			Dominant	Dominant
NMB (SAR)			SAR 56,283	SAR 51,413
NMB (USD)			$15,009	$13,710

CI, credible interval; CS, clinical surveillance; ICER, incremental cost-effectiveness ratio; NMB, net monetary benefit; QALY, quality-adjusted life year; SAR, Saudi Arabia Riyal; TAVI, transcatheter aortic valve implantation; USD, United States Dollars.

All SAR values were converted to USD using an online currency converter on 28 April 2026 (31). The conversion rate was 1.00 SAR to 0.26̅ USD.

**Figure 2:**
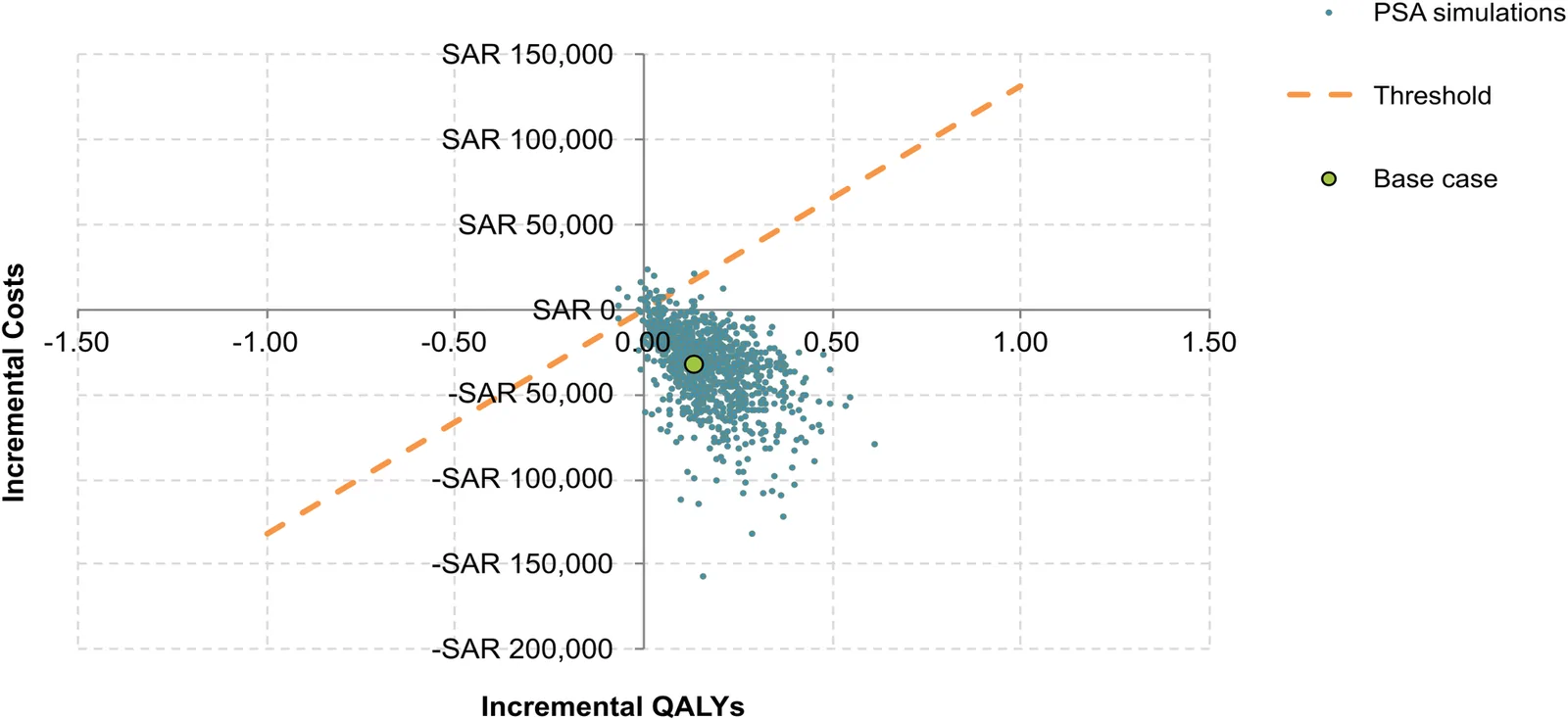
Cost-effectiveness plane. Threshold: SAR 131,706 per QALY. PSA, probabilistic sensitivity analysis; QALY, quality-adjusted life year; SAR, Saudi Arabia Riyals.

Over a lifetime horizon and compared with CS, TAVI demonstrated an increase in median survival of 0.17 years per person (Table 4). This was accompanied by a reduction of 9 strokes per 100 people (disabling and non-disabling; −0.09 per person), 12 HFH per 100 people (−0.12 per person), and 3 permanent pacemaker implantations per 100 people (−0.03 per person) over a lifetime. In addition, TAVI was associated with a lifetime increase of four reinterventions per 100 people (0.04 per person). The key driver of the results is the weighting of a disabling stroke applied to the CS arm, followed by the discounting for costs and the weighting of a disabling stroke for the TAVI arm (Supplementary Figure 4).

**Table 4:** Clinical events over a lifetime (per person, deterministic).

Outcome	TAVI	CS	Incremental
Disabling strokes	0.04	0.08	−0.04
Non-disabling strokes	0.06	0.11	−0.05
HFH	0.13	0.25	−0.12
Reinterventions	0.17	0.13	0.04
New permanent pacemakers	0.06	0.08	−0.03
Median survival (years)	10.25	10.08	0.17

CS, clinical surveillance; HFH, heart failure-related hospitalisation; TAVI, transcatheter aortic valve implantation.

Consistent with the model base case, all scenarios established TAVI as the dominant intervention compared with CS (Table 5). Scenarios incorporating alternative reintervention data identified minimal changes to the incremental cost and net monetary benefit. However, both mortality scenarios noticeably increased the net monetary benefit, and the trial-based mortality scenario yielded a substantial increase in all outcomes compared with the base case. Furthermore, the alternative time horizon scenario (five years) resulted in a small life-year gain (0.003), with TAVI found to be cost saving at 5 years due to fewer events occurring (e.g., stroke, HFH). In addition, the delayed CS AVR scenario was found to reduce the net monetary benefit compared with the base case.

**Table 5:** Scenario analysis results (deterministic).

Scenario	Inc. cost (SAR/USD)	Inc. QALYs	Inc. LYs	ICER	NMB (SAR/USD)
Base case	−33,317/−8,885	0.14	0.11	Dominant	51,413/13,710
Stroke and HFH event rates equal across arms for 5+ years	−17,955/−4,788	0.07	0.05	Dominant	26,885/7,169
Equal proportions receiving a new permanent pacemaker implant (8.4% across arms)	−29,701/−7,920	0.14	0.11	Dominant	47,798/12,746
Reintervention data: Baron et al. (47)	−34,371/−9,166	0.14	0.11	Dominant	52,467/13,991
Reintervention data: S3UR valve (46)	−34,544/−9,212	0.14	0.11	Dominant	52,640/14,037
Mortality: constant annual mortality post 65 (Male: 53.6 deaths per 1,000 people; Female 40.9 deaths per 1,000 people)	−58,788/−15,677	0.19	0.12	Dominant	83,543/22,278
All-cause mortality: trial-based (22)	−59,215/−15,791	0.57	0.64	Dominant	133,921/35,712
Five-year time horizon	−16,621/−4,432	0.01	0.003	Dominant	18,312/4,883
Delayed AVR in the CS arm (6 months)^a^	−30,795/−8,212	0.14	0.11	Dominant	48,891/13,038

CS, clinical surveillance; HFH, heart failure-related hospitalisation, ICER, incremental cost-effectiveness ratio; Inc, incremental; LYs, life years; NMB, net monetary benefit; QALYs, quality-adjusted life years; S3UR, SAPIEN 3 Ultra Resilia; USD, United States Dollars.

a This scenario only impacts costs; the clinical data remain unchanged. All SAR values were converted to USD using an online currency converter on 28 April 2026, except for the delayed AVR scenario, which was converted to USD on 23 September 2026 (31). The conversion rate was 1.00 SAR to 0.26̅ USD.

Threshold analyses demonstrated that TAVI remained cost-effective, maintaining a positive net monetary benefit across all tested variations in AVR reintervention rate (CS arm), acute and ongoing disabling stroke costs, and monthly permanent pacemaker costs (Supplementary Figures 5–8).

## 4 Discussion

Although cost-effectiveness analyses of TAVI versus CS in an asymptomatic population have recently been reported in the US and Europe, the present study provides the first assessment from the perspective of the Saudi healthcare system. Its value lies not only in confirming the economic attractiveness of early TAVI but also in demonstrating its relevance within a rapidly ageing population characterised by increasing valvular heart disease burden, documented under-diagnosis, treatment gaps, and ongoing healthcare transformation initiatives. With the advancing maturity of HTA in the KSA, utilising locally adapted cost-effectiveness data is increasingly essential to facilitate timely access and inform policy changes. By incorporating Saudi-specific costs, mortality, and resource-use inputs, this analysis provides locally relevant evidence to inform national policy, HTA, and future resource allocation decisions regarding the management of aSAS.

The evaluation identified TAVI as a dominant strategy compared with CS, with a 97.3% probability of cost-effectiveness at a WTP threshold of SAR 131,706. The economic analysis estimated that implementing TAVI in this population leads to lower costs and increased QALYs compared with CS over a lifetime horizon: a dominant result. While the TAVI arm incurred higher upfront costs due to the cost of AVR procedures and reinterventions, these were offset due to the reduced costs associated with fewer incremental strokes and HFH. The scenario analyses further reinforced the base case results by yielding similar conclusions. TAVI remained dominant when the disabling stroke and HFH event probabilities were set equal to the TAVI arm beyond 5 years (reducing the events in the CS arm). Moreover, the scenarios that implemented alternative data sources for the reintervention rate indicated a minimal but favourable incremental cost reduction and increased net monetary benefit. The threshold analyses further demonstrated the robustness of the model conclusions.

The cost-effectiveness observed within these findings is consistent with that established in Généreux et al. (23). The US evaluation identified TAVI as an economically dominant strategy for those with aSAS compared with CS, generating an ICER of approximately −$36,713 per QALY gained (23). In addition, the US analysis reported that TAVI became cost saving around the third year, similar to what was observed in this analysis, where TAVI became cost saving around the fourth year (23). Further, these findings correspond with a previous European study reporting the dominance of TAVI in an aSAS population compared with CS. Généreux et al. reported TAVI to be to be a dominant strategy across nine European countries (24).

One noticeable outcome was observed within the findings, with the number of reinterventions in the TAVI arm being higher than that seen in the CS arm. This was anticipated due to the TAVI cohort being subjected to an extended exposure time as individuals received TAVI during the first model cycle. In addition, those in the TAVI arm would be likely to have an increased chance of survival, resulting in further reintervention, dependent on valve deterioration. Although the long-term durability of TAVI valves (post-10 years) remains uncertain, the strategy remains dominant when reintervention rates are varied in scenario analyses.

The deterministic sensitivity analysis identified that the main driver of the cost-effective results was the weighting of disabling stroke in the CS arm. This is predominantly because of the increased costs and diminished benefits associated with disabling strokes. Another driver of the results was the annual discount rate applied to costs. Costs incurred in subsequent years over the lifetime horizon undergo cumulative discounting and therefore increase the influence on their present value. Nevertheless, when the discount rates are varied, the results indicate a positive net monetary benefit and remain cost-effective.

A primary strength of this economic analysis is that robust data from EARLY TAVR were used to inform the efficacy of TAVI in an aSAS population, thereby enhancing the reliability of the results (22). Multiple model inputs reflected the clinical trial, including the demographic characteristics, clinical event rates, and peri-procedural event rates. In addition, the majority of unit costs were informed by existing literature, publicly available databases, and validated stakeholder opinion (50, 52). All costs were inflated to the most recent cost year.

A further strength is that the mortality extrapolation process was conducted using non-linear methods to accurately capture the inherently non-linear relationship between covariates. Although these rates remain an approximation, this approach captured the accelerating risk gradient associated with an ageing population.

This analysis consists of several limitations that should be acknowledged. The structural limitations of the model have been outlined in previous work (23, 24). One key structural limitation to reiterate is the memoryless nature of the Markov model, which prevents tracking of the number of cycles passed since the index AVR. Consequently, the probability of reintervention in the CS arm had to be assumed constant, rather than increasing over time as observed in real-world practice. This simplification may misestimate the timing and costs of downstream reinterventions, introducing uncertainty into the model results.

A further limitation is that uncertainty inherently exists in lifetime projections due to extrapolation beyond the median follow-up of the EARLY TAVR trial. However, scenario analysis demonstrates that TAVI remains dominant even at a shorter, 5-year time horizon.

In addition, it was assumed that the outcomes of the EARLY TAVR clinical trial were generalisable to the KSA population. The direct transferability of event rates and the time from development of an indication for AVR to the actual procedure are uncertain. The trial population captured participants who were 65 years of age or above, eligible for transfemoral TAVI, had a low surgical risk, and received a balloon-expandable valve (SAPIEN 3/SAPIEN 3 Ultra valve) (21, 22). However, KSA demographic and epidemiological characteristics warrant consideration when interpreting these findings. The KSA is associated with a rapidly ageing population, projected to increase by 43% by 2050, alongside substantial multimorbidity, including a diabetes prevalence of 23.1% (59–62). High rates of diabetes and associated autonomic neuropathy in real-world cohorts can influence symptom presentation and disease progression (61, 63). This is further compounded by asymptomatic status and increased severity due to autonomic neuropathy in this specific population (61). Although these KSA-specific clinical dynamics were not explicitly parameterised in the model, higher underlying multimorbidity in practice could theoretically alter event rates for both treatment strategies. Future real-world registry studies within KSA will be valuable to evaluate whether local patient complexity modifies the observed comparative cost-effectiveness of TAVI.

Further, in the EARLY TAVR trial, the median wait time to AVR following decision to treat was 32 days, with clinical events occurring captured through the trial-derived event rates applied over the model horizon (22). However, real-world clinical practice often involves longer health system wait times for surgical valve replacement (64, 65). Extended pre-procedural delays increase the probability of clinical events (22, 66). In the KSA, the effectiveness of clinical surveillance is likely to be reduced due to low utilisation of AVR, reflected by the number of AVR procedures conducted compared with the estimated prevalence of AS, combined with the apparent lack of designated valve clinics, particularly in peripheral hospitals (19, 67, 68). Therefore, the trial-informed estimates may not fully represent TAVI's relative clinical and economic benefit.

A final limitation pertains to the paucity of KSA-specific data for population norms and cost-effectiveness measures (namely, HRQoL population norms, discount rates, and WTP threshold). UK EQ-5D population norms were applied in the model, under the assumption that the data were reflective of a KSA population, which may not be the case. In the absence of KSA-specific HTA guidelines, approximations were implemented concerning the discounting factors, of which proxies were applied accordingly, and the WTP threshold, which was derived using the GDP per capita. It should be noted that because TAVI was established as a dominant intervention, the threshold does not impact the conclusion yielded from this study.

## 5 Conclusion

In the KSA, TAVI is a procedural approach for individuals with aSAS at low procedural risk, and if implemented into policy and practice, could result in cost savings on the KSA health budget. These findings support policies and the growing body of evidence promoting the early detection of AS and timely intervention before symptomatic presentation, as opposed to providing CS with a delayed AVR procedure. Although this model estimates the potential economic advantage of an early interventional strategy as evaluated in EARLY TAVR, establishing the precise optimal timing for intervention remains an important area for future research and would further support the endorsement of implementing TAVI in a low-risk aSAS population in the KSA.

## Data Availability

The original contributions presented in the study are included in the article/Supplementary Material; further inquiries can be directed to the corresponding author.
