# Supplementary material for: Cost-effectiveness of transcatheter aortic valve implantation for asymptomatic severe aortic stenosis in the Kingdom of Saudi Arabia
Source: Front Cardiovasc Med. 2026 Oct 29;13:1937664. doi: 10.3389/fcvm.2026.1937664 (PMC13757413; doi:10.3389/fcvm.2026.1937664)
Supplement: Supplementary file 1 [file Supplementaryfile1.docx]

Supplementary Material

# 1 Supplementary Figures

**Supplementary Figure 1:** Time to conversion to AVR in the clinical surveillance (CS) arm

The five-year cumulative proportion of the CS arm converting to AVR was sourced from KM data from the EARLY TAVR trial and was digitised (1). The KM data was then extrapolated beyond the five-year trial period using an exponential distribution. Within the lifetime horizon, 100% of those alive in the CS arm converted to AVR. *AVR* aortic valve replacement, *KM* Kaplan-Meier, *S(t)* survival.

**Supplementary Figure 2:** Cost-effectiveness acceptability curve

Quality-adjusted life year, *SAR* Saudi Arabia Riyals, *TAVI* transcatheter aortic valve implantation.

**Supplementary Figure 3:** Deterministic cost breakdown by category (per person)

*AVR* aortic valve replacement, *PPM* permanent pacemaker, *SAR* Saudi Arabia Riyals, *TAVI* transcatheter aortic valve implantation.

**Supplementary Figure 4:** Tornado diagram

*AVR* aortic valve replacement, *CS* clinical surveillance, *NMB* net monetary benefit, *PPM* permanent pacemaker, *SAR* Saudi Arabia Riyals.

**Supplementary Figure 5:** Threshold Analysis for AVR reintervention (CS arm)

*AVR* aortic valve replacement, *CS* clinical surveillance, *SAR* Saudi Arabia Riyals.

**Supplementary Figure 6:** Threshold analysis for ongoing disabling stroke cost

*SAR* Saudi Arabia Riyals.

**Supplementary Figure 7:** Threshold analysis for acute disabling stroke cost

*SAR* Saudi Arabia Riyals.

**Supplementary Figure 8:** Threshold analysis for ongoing permanent pacemaker cost

*SAR* Saudi Arabia Riyals.

# 2 Supplementary Tables

**Supplementary Table 1:** AIC and BIC values for the time to conversion to AVR in the CS arm

| **Independent statistical model distributions** | **Akaike’s Information Criteria (AIC)** | **Bayesian Information Criteria (BIC)** |
| --- | --- | --- |
| Exponential | **1183.23** | **1190.32** |
| Generalised gamma | 1185.34 | 1192.44 |
| Gompertz | 1185.02 | 1195.66 |
| Log-logistic | 1199.76 | 1203.31 |
| Log-normal | 1199.90 | 1206.99 |
| Weibull | 1200.95 | 1208.04 |

Exponential rate coefficient = -2.95. Cholesky parameter estimate = 0.08.

**Supplementary Table 2:** Base case and scenario data for the reintervention of aortic valve replacement

|  | **Leon et al. (2025)** **(2)** **Bourguignon et al. (2015) (3)**  **Base Case Annual Probability** | **Baron et al. (2024) (4)**  **Scenario Annual Rate** | **SAPIEN 3 Ultra RESILIA (5)**  **Scenario Annual Rate** |
| --- | --- | --- | --- |
| **Year 1** | 0.48% | 0.36% | 0.00% |
| **Year 2** | 0.44% | 0.17% | 0.00% |
| **Year 3** | 0.59% | 0.13% | 0.00% |
| **Year 4** | 0.59% | 0.19% | 0.00% |
| **Year 5** | 0.59% | 0.26% | 0.34% |
| **Year 6** | 1.12% | 0.41% | 0.76% |
| **Year 7** | 1.12% | 0.60% | 0.76% |
| **Year 8** | 1.12% | 0.51% | 0.76% |
| **Year 9** | 1.23% | 0.75% | 0.76% |
| **Year 10** | 1.48% | 0.59% | 0.76% |
| **Year 11** | 1.92% | 0.62% | 0.76% |
| **Year 12** | 2.19% | 0.64% | 0.76% |
| **Year 13** | 2.56% | 0.67% | 0.76% |
| **Year 14** | 3.14% | 0.69% | 0.76% |
| **Year 15** | 3.67% | 0.72% | 0.76% |
| **Year 16** | 4.41% | 0.74% | 0.76% |
| **Year 17** | 4.99% | 0.77% | 0.76% |
| **Year 18** | 5.85% | 0.79% | 0.76% |
| **Year 19** | 6.11% | 0.82% | 0.76% |
| **Year 20** | 6.42% | 0.85% | 0.76% |
| **Year 21** | 6.95% | 0.85% | 0.76% |
| **Year 22** | 7.24% | 0.85% | 0.76% |
| **Year 23+** | 7.24% | 0.85% | 0.76% |

**Supplementary Table 3:** Probability sensitivity analysis distributions

| Parameter | DSA Variance | PSA Distribution |
| --- | --- | --- |
| Proportion of strokes that are disabling | +/- 20% | Beta. 20% SE assumed |
| Proportion receiving a permanent pacemaker | +/- 20% | Beta (alpha = no. with PPM, beta = no. without PPM from EARLY TAVR Table S8) (1) |
| Stroke probability: Year 1 | +/- 20% | Beta (alpha = no. with stroke, beta = no. without stroke from EARLY TAVR) (1) |
| Stroke probability: Year 2 | +/- 20% | Beta (alpha = no. with stroke, beta = no. without stroke from EARLY TAVR) (1) |
| Stroke probability: Year 3 | +/- 20% | Beta (alpha = no. with stroke, beta = no. without stroke from EARLY TAVR) (1) |
| Stroke probability: Year 4 | +/- 20% | Beta (alpha = no. with stroke, beta = no. without stroke from EARLY TAVR) (1) |
| Stroke probability: Year 5+ | +/- 20% | Beta (alpha = no. with stroke, beta = no. without stroke from EARLY TAVR) (1) |
| HFH probability: Year 1 | +/- 20% | Beta (alpha = no. with HFH, beta = no. without HFH from EARLY TAVR) (1) |
| HFH probability: Year 2 | +/- 20% | Beta (alpha = no. with HFH, beta = no. without HFH from EARLY TAVR) (1) |
| HFH probability: Year 3 | +/- 20% | Beta (alpha = no. with HFH, beta = no. without HFH from EARLY TAVR) (1) |
| HFH probability: Year 4 | +/- 20% | Beta (alpha = no. with HFH, beta = no. without HFH from EARLY TAVR) (1) |
| HFH probability: Year 5+ | +/- 20% | Beta (alpha = no. with HFH, beta = no. without HFH from EARLY TAVR) (1) |
| Time period in which mortality doubles (7 years) | +/- 20% | Log-normal (Ln SE =0.13, based on assumed 95% CI of 6 and 10 years). |
| Disabling stroke (month 1) mortality relative risk | +/- 20% | Log-normal. SE assumed 20% of the mean. |
| Non-disabling stroke (month 1) mortality relative risk | +/- 20% | Log-normal. SE assumed 20% of the mean. |
| Disabling stroke (month 2+) mortality relative risk | +/- 20% | Log-normal. SE assumed 20% of the mean. |
| Non-disabling stroke (month 2+) mortality relative risk | +/- 20% | Log-normal. SE assumed 20% of the mean. |
| HFH mortality relative risk | +/- 20% | Log-normal. SE assumed 20% of the mean. |
| Echocardiogram (unit cost) | +/- 20% | Gamma. SE assumed 20% of the mean. |
| Consultation (unit cost) | +/- 20% | Gamma. SE assumed 20% of the mean. |
| Disabling stroke (initial cost) | +/- 20% | Gamma. SE assumed 20% of the mean. |
| Disabling stroke (ongoing cost per year) | +/- 20% | Gamma. SE assumed 20% of the mean. |
| Non-disabling stroke (initial cost) | +/- 20% | Gamma. SE assumed 20% of the mean. |
| Non-disabling stroke (ongoing cost per year) | +/- 20% | Gamma. SE assumed 20% of the mean. |
| HFH general ward stay | +/- 20% | Gamma. SE assumed 20% of the mean. |
| HFH ICU/CCU stay | +/- 20% | Gamma. SE assumed 20% of the mean. |
| HFH consultation | +/- 20% | Gamma. SE assumed 20% of the mean. |
| HFH CVI consultation | +/- 20% | Gamma. SE assumed 20% of the mean. |
| HFH: MCS procedure | +/- 20% | Gamma. SE assumed 20% of the mean. |
| PPM (implantation cost) | +/- 20% | Gamma. SE assumed 20% of the mean. |
| PPM (ongoing cost per month) | +/- 20% | Gamma. SE assumed 20% of the mean. |
| TAVI: Cost of intervention | +/- 20% | Gamma. SE assumed 20% of the mean. |
| SAVR: Cost of intervention (complex) | +/- 20% | Gamma. SE assumed 20% of the mean. |
| Echocardiogram (per year) | +/- 20% | Gamma. SE assumed 20% of the mean. |
| Consultation (per year) | +/- 20% | Gamma. SE assumed 20% of the mean. |
| HFH general ward stay (length of stay, days) | +/- 20% | Gamma. SE assumed 20% of the mean. |
| HFH CCU stay (length of stay, days) | +/- 20% | Gamma. SE assumed 20% of the mean. |
| HFH CVI consultation | +/- 20% | Gamma. SE assumed 20% of the mean. |
| HFH MCS procedure (proportion requiring) | +/- 20% | Beta. SE assumed 20% of the mean. |
| Alive-and-well utility input | +/- 20% | Beta. SE assumed 20% of the mean. |
| Non-disabling stroke utility input | +/- 20% | Beta. SE assumed 20% of the mean. |
| Disabling stroke (month 1) utility input | +/- 20% | Beta. SE assumed 20% of the mean. |
| Disabling stroke (month 2+) utility input | +/- 20% | Beta. SE assumed 20% of the mean. |
| HFH utility input | +/- 20% | Beta. SE assumed 20% of the mean. |

*CCU* critical care unit, *CVI* cardiovascular imaging, *HFH* heart failure-related hospitalisation, *MCS* mechanical circulatory support, *NCC* National Cost Collection, *NICE* National Institute for Health and Care Excellence, *PPM* permanent pacemaker, *PSA* probabilistic sensitivity analysis, *SAVR* surgical aortic valve replacement, *TAVI* transcatheter aortic valve implantation.

**Supplementary Table 4:** External validation of model vs. EARLY TAVR

| **TAVI Arm - Stroke** | | | | | | |
| --- | --- | --- | --- | --- | --- | --- |
| **Time (years)** | **Rate of stroke (model)** | | | **Cumulative rate (model)** | **EARLY TAVR KM estimates** | |
| 1 | 1.32% | | | 1.32% |  | |
| 2 | 1.34% | | | 2.64% | 2.7% | |
| 3 | 1.35% | | | 3.96% |  | |
| 4 | 0.69% | | | 4.62% |  | |
| 5 | 0.92% | | | 5.49% | 5.4% | |
| **CS Arm - Stroke** | | | | | | |
| **Time (years)** | **Rate of stroke (model)** | | | **Cumulative rate (model)** | **EARLY TAVR KM estimates** | |
| 1 | 1.79% | | | 1.8% |  | |
| 2 | 2.05% | | | 3.8% | 3.7% | |
| 3 | 1.86% | | | 5.6% |  | |
| 4 | 1.90% | | | 7.4% |  | |
| 5 | 2.18% | | | 9.4% | 9.5% | |
| **TAVI Arm – Heart-failure hospitalisation (HFH)** | | | | | | |
| **Time (years)** | | **Rate of HFH (model)** | | **Cumulative rate (model)** | **EARLY TAVR KM estimates** | |
| 1 | | 1.32% | | 1.3% |  | |
| 2 | | 0.22% | | 1.5% | 1.6% | |
| 3 | | 1.34% | | 2.9% |  | |
| 4 | | 1.36% | | 4.2% |  | |
| 5 | | 1.38% | | 5.6% | 5.3% | |
| **CS Arm – HFH** | | | | | | |
| **Time (years)** | | **Rate of HFH (model)** | | **Cumulative rate (model)** | **EARLY TAVR KM estimates** | |
| 1 | | 5.16% | | 5.2% |  | |
| 2 | | 2.84% | | 8.0% | 7.8% | |
| 3 | | 1.46% | | 9.5% |  | |
| 4 | | 0.99% | | 10.4% |  | |
| 5 | | 2.24% | | 12.7% | 12.0% | |
| **Time (months)** | | **CS trial cohort converted to AVR** | | | **EARLY TAVR KM estimates** |  |
|  |  | **n** | **%** | |  |  |
| 6 | | 120 | 27.0% | | 26.2% |  |
| 12 | | 208 | 47.0% | | 47.2% |  |
| 24 | | 319 | 71.4% | | 71.4% |  |
| 36 | | 378 | 84.7% | | 86.1% |  |
| 48 | | 410 | 91.8% | | 90.4% |  |
| 60 | | 427 | 95.6% | | 95.2% |  |

*Continued on next page*

| **Time (months)** | **Cumulative proportion dead (model - used in a scenario analysis only)** | | **EARLY TAVR KM estimates** | |
| --- | --- | --- | --- | --- |
|  | **CS** | **TAVI** | **CS** | **TAVI** |
| 6 | 0.5% | 0.5% | 1.1% | 1.1% |
| 12 | 1.4% | 1.4% | 1.3% | 1.3% |
| 24 | 3.8% | 3.6% | 2.9% | 3.8% |
| 36 | 6.7% | 6.2% | 7.2% | 5.3% |
| 48 | 10.1% | 9.1% | 9.8% | 8.2% |
| 60 | 13.7% | 12.2% | 13.8% | 13.6% |

*AVR* aortic valve replacement, *CS* clinical surveillance, *HFH* heart failure-related hospitalization, *KM* Kaplan-Meier, *TAVI* transcatheter aortic valve implantation, *TAVR* transcatheter aortic valve replacement

# 3 References

1.	Généreux P, Schwartz A, Oldemeyer JB, Pibarot P, Cohen DJ, Blanke P, et al. Transcatheter Aortic-Valve Replacement for Asymptomatic Severe Aortic Stenosis. The New England Journal of Medicine. 2025;392(3):217–27.doi.10.1056/NEJMoa2405880

2.	Leon MB, Mack MJ, Pibarot P, Hahn R, Thourani VH, Kodali SH, et al. Transcatheter or Surgical Aortic-Valve Replacement in Low-Risk Patients at 7 Years. The New England Journal of Medicine. 2025;394(8):773–83.doi.10.1056/NEJMoa2509766

3.	Bourguignon T, Bouquiaux-Stablo A-L, Candolfi P, Mirza A, Loardi C, May M-A, et al. Very long-term outcomes of the Carpentier-Edwards Perimount valve in aortic position. The Annals of Thoracic Surgery. 2015;99(3):831–7.doi.10.1016/j.athoracsur.2014.09.030

4.	Baron SJ, Ryan MP, Chikermane SG, Thompson C, Clancy S, Gunnarsson CL. Long-term risk of reintervention after transcatheter aortic valve replacement. American Heart Journal. 2024;267:44–51.doi.10.1016/j.ahj.2023.10.002

5.	Kaneko T, Johnston D, Bavaria JE, Thourani V, Lifesciences. E. Propensity-matched 8-year Outcomes Following Surgical Aortic Valve Replacement With Novel Calcification-resistant Versus Contemporary Tissue Bioprostheses (abstract). The Heart Valve Society. 2025.
